# Understanding the impact of area-based interventions on area safety in deprived areas: realist evaluation of a neighbour nuisance intervention in Arnhem, the Netherlands

**DOI:** 10.1186/s12889-016-2905-4

**Published:** 2016-03-31

**Authors:** Daniëlle Kramer, Janneke Harting, Anton E. Kunst

**Affiliations:** Department of Public Health, Amsterdam Medical Centre (AMC), University of Amsterdam, PO Box 22660, 1100 DD Amsterdam, The Netherlands

**Keywords:** Safety, Disorder, Area deprivation, Realist evaluation, Intervention

## Abstract

**Background:**

Area-based health inequalities may partly be explained by higher levels of area disorder in deprived areas. Area disorder may cause safety concerns and hence impair health. This study assessed how, for whom and in what conditions the intervention Meeting for Care and Nuisance (MCN) had an impact on neighbour nuisance and area safety in four deprived districts in Arnhem, the Netherlands.

**Methods:**

Realist evaluation methodology was applied to uncover how, for whom, and under what conditions MCN was expected to and actually produced change. Expected change was based on action plans and scientific theories. Actual change was based on progress reports, media articles, interviews with district managers, and quantitative surveys.

**Results:**

Three levels of impact were distinguished. At the organisational level, partly as expected, MCN’s coordinated partnership strategy enabled role alignment, communication, and leadership. This resulted in a more efficient approach of nuisance households. At the level of nuisance households, as expected, MCN’s joint assistance and enforcement strategy removed many of the underlying reasons for nuisance. This resulted in less neighbour nuisance. At the district level, perceptions of social control and area safety improved only in one district. Key conditions for change included a wider safety approach, dense population, and central location of the district within the city.

**Conclusions:**

This realist evaluation provided insight into the mechanisms by which a complex area-based intervention was able to reduce neighbour nuisance in deprived areas. Depending on wider conditions, such a reduction in neighbour nuisance may or may not lead to improved perceptions of area safety at the district level.

## Background

There is ample evidence of differences in health between deprived and non-deprived areas. Residents of deprived areas report worse health than those in non-deprived areas [1–4]. Several studies suggest that these health inequalities can partly be explained by different levels of area disorder [5–9]. Residents of deprived areas perceive more physical disorder (e.g. litter, graffiti) and social disorder (e.g. nuisance from neighbours or youth) than residents of non-deprived areas [5, 7, 8]. Area disorder may cause people to feel unsafe, which may negatively affect their health by increasing stress, anxiety, physical inactivity, or social exclusion [10].

A review has shown that, numerous area-based initiatives have been implemented in deprived areas across Western-Europe in the past decade [11]. These initiatives consist of multiple interventions that aim to tackle the various socio-economic and environmental problems in deprived areas, including physical and social disorder. Two of these area-based initiatives have been evaluated for their impact on area disorder and subsequent safety concerns. Results have been mixed. After 6 years, target areas of the English New Deal for Communities had significantly larger reductions in perceived lawlessness and dereliction than other deprived areas [12]. There were no differences in reductions of fear of crime and feeling unsafe after dark. After 5 years, target areas of the English Single Regeneration Budget saw larger reductions in the number of residents feeling very unsafe than the rest of England [13]. However, differences were small and not tested for significance. There were no differences in reductions of disorder such as vandalism, loose dogs, and litter.

These quantitative evaluation studies have been criticized for their lack of attention to mechanisms of change and programme theory [14–16]. To improve future initiatives, research needs to extend its attention from outcomes towards the processes leading to these outcomes. Pawson and Tilley’s [17] realist evaluation methodology offers a useful approach to understand the inner workings of complex initiatives. The realist methodology aims to uncover how an intervention works, for whom, and under what conditions. More specifically, it tries to identify the so-called mechanisms of change. Mechanisms refer to individuals’ responses triggered by the intervention that lead to change. These mechanisms will only be activated under certain conditions. Interventions are often based on assumptions about possible mechanisms and conditions, but these so called programme theories are rarely made explicit. A key purpose of the realist methodology is to identify these programme theories, and to try and refine it using evidence on how the interventions worked in practice.

Only few studies have applied the realist methodology to understand how area-based interventions have influenced area disorder and safety concerns. A good example is that of Nanninga and Glebbeek [18]. They explored whether and how two new sports fields had an impact on nuisance from youth and related crime in the Netherlands. Anticipated mechanisms of change included ‘boredom reduction’ and ‘role modelling’. Police records showed that since the arrival of the sports fields, there were less reports of nuisance, but more reports of crime. The drop in nuisance could be explained by the anticipated ‘boredom reduction’ mechanism and the newfound ‘confrontation reduction’ mechanism.

Little is known about how area disorder and safety are affected by area-based interventions that are oriented at households. A pioneering intervention in this field is the Dundee Families Project of 1996 [19]. This project aimed to help families that were evicted or at risk of eviction because of neighbour nuisance. A mix of counselling, family support, surveillance, and regulations was offered to families, either by reaching out to families in their own homes or by admitting families to a temporary home and supporting them there. In the latter case, families could either be admitted to a core residential unit with up to four other families that were part of the project, or to one of the dispersed flats run by the project. An evaluation study revealed that the intervention was able to reduce nuisance caused by these families [19]. Unfortunately, the study did not explore how, for whom or under what conditions this intervention was able to reduce neighbour nuisance and safety concerns.

An opportunity to acquire such knowledge arose with the introduction of the Meeting for Care and Nuisance (MCN) (*Overleg Zorg en Overlast*) in the four most deprived districts of Arnhem, a mid-sized city in the east of the Netherlands. This intervention is part of a large area-based initiative that was implemented in 2008 in the forty most deprived districts of the Netherlands, including the four deprived districts in Arnhem. MCN aimed to reduce neighbour nuisance (e.g. loud music, fights, neglected property), which was regarded to be the main cause of perceived unsafety in these areas. The current study aimed to explore how, for whom, and under what conditions MCN had an impact on neighbour nuisance and area-level safety in the four target districts. Following the realist methodology, we first identified the programme theory on how MCN was anticipated to work. Then, we used this programme theory as a guide to assess how MCN actually worked.

## Methods

### Design

An embedded case study design was applied [20]. Each of the four deprived target districts in Arnhem represented a separate case. Within each case, three levels of analysis were distinguished: 1) the organisations that were involved in the implementation of MCN, 2) the households that were causing nuisance, 3) the four deprived districts at large.

### The intervention

Neighbour nuisance is addressed by MCN in different steps. To start with, each district holds monthly meetings with the police, local housing corporations, the care coordinator, and the district manager. Under the leadership of the district manager, organisations exchange information about new and existing nuisance households during the meetings. New households are included based on signals of nuisance reported by the participating organisations, neighbours, care professionals, or others. For each new nuisance household, the care coordinator develops a plan of action that specifies what needs to be done and which organisation is responsible for what action. Plans of action are discussed with the organisations during the monthly meetings. When plans are agreed upon, the households are paid a visit by the care coordinator and a representative of one of the other organisations. The care coordinator discusses the plan of action with the household. The household’s vision on the plan is incorporated in a guidance agreement that specifies what the household should do to reduce the nuisance (e.g. turning down their music after 10 p.m). Usually, the guidance agreement is accompanied with assistance from care professionals, as many of the households deal with unresolved underlying problems (e.g. debt, addiction, psychiatric illness, unemployment, or neglect). For households who have received assistance in the past, such assistance can be conditional (e.g. households first have to clean their front yard in order to receive debt assistance). If households are unwilling to cooperate, their case is forwarded to the justice department, who decides on whether households will be threatened with sanctions such as eviction or benefit reduction. Cases are closed when no new nuisance signals are reported. During the entire process, the care coordinator monitors the progress of the households and organisations.

### The cases

MCN was first implemented in 2006 in district 1. This district is a working-class district characterized by low levels of social cohesion and trust in the municipality. It has a long history of social problems and neighbour conflicts. Many repressive and restructuring interventions have been implemented in the past, but without success. In 2010, MCN was extended to districts 2 to 4. District 2 is a centrally located and densely populated district that is characterized by high levels of creative enterprises and students. Problems related to drug users, criminal activities, and deterioration of public spaces have prompted past interventions that have been somewhat successful. Districts 3 and 4 are both post-World War II districts that are characterised by uniform low-quality housing occupied by people of low socio-economic status and starters at the housing market. Various restructuring and social interventions have been implemented in the past, but with limited success. More information about the characteristics of the four districts can be found in Table 1 [21]. As district 3 covers a relatively large and diverse area, characteristics and results are described separately for three different parts of this district.

**Table 1:** Characteristics of the districts and its residents [21]

District	Number of residents	Size (acres)	Population density (number of residents per km^2^)	Privately owned houses (%)	Mean income per resident (€)	Residents of non-western origin (%)
Target district 1	5825	510	1340	28 %	16500	33 %
Target district 2	7250	69	10581	28 %	14200	25 %
Target district 3a	4505	427	1456	36 %	17000	38 %
Target district 3b	5570	172	3705	44 %	18200	26 %
Target district 3c	7180	135	5327	18 %	13900	42 %
Target district 4	8175	143	5727	20 %	18000	45 %
City of Arnhem	148070	10154	1511	43 %	21000	18 %

### Data collection

Based on the principles of the realist evaluation, data collection was iterative and included a wide range of sources (Table 2). Two types of qualitative evidence were obtained to assess how MCN was expected to work, i.e. the programme theory underlying MCN. First, we searched the web for action plans that set out how policy makers expected MCN to work. Five action plans were included. Second, we searched the literature for scientific theories that complemented the expectations set out in the action plans.

**Table 2:** Data sources

CONTENT	AUTHOR	NAME	YEAR	DISTRICT			
				1	2	3	4
Documents							
Action plans							
Description of interventions planned for district 1–4 as part of the larger area-based initiative (incl. MCN)	Municipality of Arnhem	DOC1	2007		X		
		DOC2	2007				X
		DOC3	2007			X	
		DOC4	2007	X			
		DOC5	2007	X			
Progress reports							
Progress report of interventions in district 1–4 as part of the larger area-based initiative (incl. MCN)	Municipality of Arnhem	DOC6	2009	X	X	X	X
		DOC7	2009	X	X	X	X
		DOC8	2010	X	X	X	X
		DOC9	2010	X	X	X	X
		DOC10	2010	X	X	X	X
		DOC11	2011	X	X	X	X
		DOC12	2011	X	X	X	X
		DOC13	2011	X	X	X	X
		DOC14	2012	X	X	X	X
Progress report of household interventions (incl. MCN)	Government	DOC15	2010	X			
Progress report of MCN	Welfare organisation	DOC16	2011	X	X	X	X
		DOC17	2011	X	X	X	X
		DOC18	2012	X	X	X	X
Media reports							
Online news report about a guided tour to district 1–4 (incl. MCN)	Journalist	DOC19	2009	X	X		
		DOC20	2010	X	X	X	X
Online news report about MCN	Journalist	DOC21	2009	X			
Newspaper section about safety in Arnhem (incl. MCN)	Journalist	DOC22	2010	X	X	X	X
Magazine about Dutch social enterprises (incl. MCN)	Journalist	DOC23	2011	X			
Short video of an interview with an MCN care coordinator	Journalist	DOC24	2011			X	
Survey							
Survey about liveability, safety, neighbourhood problems, victimization, and municipal functioning as perceived by residents of Arnhem (incl. district 1–4)	Municipality of Arnhem	SURV1	2005	X	X	X	X
			2007	X	X	X	X
		SURV2	2009	X	X	X	X
		SURV3	2011	X	X	X	X
Interviews							
Interviews with the district managers of district 1–3	n.a.	INT1	2012	X			
		INT2	2013	X			
		INT3	2012	X	X	X	
		INT4	2013	X	X	X	
		INT5	2012		X		
		INT6	2013		X		

Three types of qualitative and quantitative evidence were obtained to assess how MCN actually worked. First, we searched the web for documents that reported on experiences with MCN. Search terms included “OZO”, “Overleg Zorg en Overlast”, “Zorgcoordinator”, and “Arnhem”. Seventeen progress reports and media reports were included that contained information on mechanisms, outcomes and/or contexts specific to MCN in practice.

Second, we obtained existing quantitative data on district-level perceptions of neighbour nuisance and area safety from the Survey for Liveability and Safety (2005, 2007) and the Integral Safety Monitor (2009, 2011). Both surveys aimed to assess residents’ perceptions of safety and liveability and included questions regarding overall safety perception, criminal victimization, perceived neighbourhood problems, and judgement of police and municipal government. For the Survey for Liveability and Safety, individuals of 18 years and older were asked to fill in a paper-and-pencil or web-based questionnaire. In 2005, a total of 6941 adults were approached in the four target districts, of which 1661 adults completed the survey. This resulted in a response rate of 24 %, ranging from 22 to 27 % between the four target districts. The response rate was lower than in the city of Arnhem as a whole, where the response rate was 29 %. In 2007, a total of 4433 adults (12 % of the population) were approached in the four target districts, of which 1210 adults completed the survey. This resulted in a response rate of 27 %, ranging from 24 to 29 % between the four target districts. The response rate was lower than in the city of Arnhem as a whole, where the response rate was 37 %. For the Integrated Safety Monitor, individuals of 15 years and older were asked to fill in a paper-and-pencil or web-based questionnaire, or were interviewed by telephone. In 2011, a total of 4175 individuals (11 % of the population) were approached in the four target districts, of which 1463 adults completed the survey. This resulted in a response rate of 35 % in all four target districts. The response rate was lower than in Arnhem as a whole, where the response rate was 42 %. For 2009, information about sample sizes and response rates per district were unknown.

Third, we performed two rounds of individual semi-structured interviews with the district managers of three target districts. The district manager of district 4 was unwilling to participate. The first round of interviews took place after we constructed the programme theory in September 2012. District managers were asked to confirm, falsify or refine our programme theory based on their experiences with MCN in practice. The second round of interviews took place in November 2013, after we assembled and integrated the available evidence on how MCN actually worked. District managers were asked to confirm, falsify, or refine our integration of the evidence, taking into account their own experiences with MCN. During the interviews, the district managers provided us with two extra progress reports not available on the web.

### Data analysis

Data analysis was guided by the framework approach of Ritchie and Spencer [22]. First, we articulated the programme theory. We extracted information from the action plans about the outcomes and mechanisms that were anticipated to result from MCN. These expectations were complemented with information from the scientific theories. Second, we indexed the evidence on how MCN actually worked, using the programme theory as a guide. We flagged information about the mechanisms and outcomes set out in the programme theory, but were also alert for unanticipated mechanisms, outcomes, or conditions. For each extracted piece of information, we specified the data source, year, and district (if applicable). Third, we charted the extracted information. The information was separated into three datasets: one on the mechanisms, one on the conditions, and one on the outcomes. Within each dataset, information was sorted by level of analysis: organisations, nuisance households, and districts. Finally, we mapped and interpreted the information. Recurrent patterns of information were grouped and labeled. When new patterns emerged, data sources were checked again for possible additional information. Patterns were regularly discussed with the members of the research team. Where possible, patterns were compared across cases.

The Medical Ethics Committee of the Academic Medical Centre in Amsterdam, the Netherlands, has confirmed that ethics approval is not necessary, as the Medical Research Involving Human Subjects Act (WMO) does not apply to our study.

## Results

### Programme theory

**Fig. 1:**
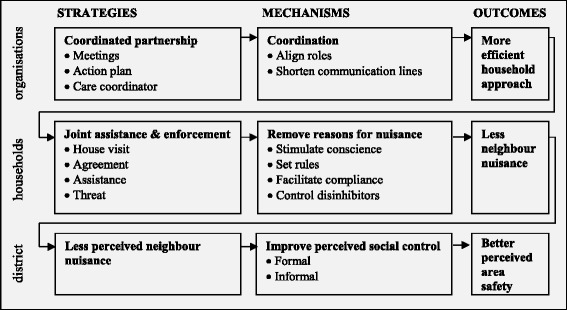
Programme theory on MCN’s strategies and anticipated mechanisms and outcomes

The programme theory specifies the mechanisms and outcomes expected to result from MCN at the levels of organisations, nuisance households, and districts (Fig. 1). Expectations were identical across the four cases. No conditions were made explicit in the action plans. The following sections specify the mechanisms and outcomes expected to result from MCN at each level, starting with the level of organisations.

#### Organisations

At the level of organisations, the partnership strategy (meetings, action plan, and care coordinator) applied by MCN was expected to improve the coordination of the actions of all participating organisations, leading to a more efficient approach of nuisance households. The action plans describe how past attempts to reduce neighbour nuisance in the four target districts were believed to have been inefficient due to a lack of coordination among organisations: “*One of the biggest problems when approaching and helping the problem households, is the bad coordination between the various organisations who are, more than incidental, working with an individual or family without being aware of each other’s work* (Document (DOC) 5, see Table 2 for more details)”. The organisational strategy applied by MCN was expected to improve coordination and hence lead to a more efficient approach of households. The type of partnership chosen by MCN fits nicely into the partnership typology of Winer and Karen [23]. They distinguish three types of partnerships: cooperative, coordinated, and collaborative. Of these, MCN belongs to the category of coordinated partnership. This type of partnership refers to a longer-term interaction around a specific effort, in this case the reduction of neighbour nuisance. It aims to increase coordination by aligning roles and by opening up communication channels. Authority remains with the organisations, which may cause power issues. In contrast, a cooperative partnership refers to a less intense short-term interaction with the aim to exchange information without any common mission, structure, or planning. On the other hand, a collaborative partnership refers to a more intense long-term interaction with the aim to create new structures with a common mission, comprehensive planning, and well-defined communication channels.

#### Nuisance households

At the level of nuisance households, the joint assistance and enforcement strategy (house visit, agreement, assistance, and threat) applied by MCN was expected to remove underlying reasons for nuisance, leading to a reduction in neighbour nuisance. The action plans describe how neighbour nuisance in the four target districts was believed to be partly due to a lack of enforcement: “*Due to a long-lasting lack of enforcement, (part of) neighbourhoods have been able to withdraw from society and some places have developed their own rules and norms* (DOC1-4)”. As many of the problem households dealt with underlying problems, it was said that enforcement needed to be combined with assistance to help solve these problems. The joint assistance and enforcement strategy applied by MCN was expected to reduce the amount of neighbour nuisance. Clarke’s situational crime prevention theory suggests this to be the result of a reduction in the underlying reasons for nuisance [24]. This theory proposes four main mechanisms to prevent crime: 1) increase perceived risk of getting caught, 2) increase perceived efforts to commit the crime, 3) reduce perceived rewards of the crime, and 4) reduce reasons for committing the crime. While the first three mechanisms are mostly applicable to hard crimes, the fourth mechanism is relevant to soft crimes like neighbour nuisance. Clarke proposes four sub mechanisms to reduce reasons: 1) stimulate conscience of the unaccepted behaviour, 2) set rules to remove any ambiguity concerning the acceptability of the behaviour, 3) control disinhibitors like alcohol or drugs, that reduce people’s capacity to manage their behaviour, and 4) facilitate compliance to the accepted behaviour. A variety of strategies can be introduced to trigger these mechanisms. While Clarke proposes various physical environmental strategies, MCN focuses on social strategies, i.e. a combination of assistance and enforcement.

#### Districts

At the level of districts, a reduction in neighbour nuisance was expected to improve perceptions of social control, leading to improved perceptions of area safety. The action plans describe how safety concerns in the four target districts were believed to be the result of soft crimes like physical disorder, disobedience of rules, and nuisance (DOC1-4). Even though nuisance was caused by only a handful of households, their behaviour was suggested to greatly affect the atmosphere and image of the district: “*People are no longer willing and able to confront others or inform organisations about what they see happening in their neighbourhood* (DOC1-4)”. A reduction in neighbour nuisance was expected to improve district-wide perceptions of area safety. The incivilities thesis endorses this hypothesis, and suggests this change to be the result of improved perceptions of social control [25]. The incivilities thesis posits that residents may interpret incivilities such as neighbour nuisance as a sign that fellow residents (informal control) and authorities (formal control) are unwilling or unable to preserve order in their neighbourhood. This perceived lack of informal and formal social control may cause residents to feel personally at risk of serious crime.

### Evidence on how MCN actually worked

The next sections describe the mechanisms and outcomes actually resulting from MCN, and the conditions under which they were activated. Results will be discussed consecutively for the levels of organisations, nuisance households, and districts.

#### Organisations

##### Mechanisms

(I) Align roles
  In line with the programme theory, the coordinated partnership strategy was mentioned to increase the amount of role alignment among the participating organisations (DOC9,13,16–18,21–24; Interview (INT) 1, see Table 2 for more details). Because of the alignment, each organisation knew what was expected from them. A programme manager mentioned: *“there is a much clearer picture of actions, responsibilities, and tasks* (DOC16)”.
(II) Shorten communication lines
  In line with the programme theory, the coordinated partnership strategy was found to shorten the lines of communication between organisations in three ways (DOC16–18). First, the monthly meetings enabled organisations to familiarize with each other, making it easier for them to contact each other outside the meetings. Third, the care coordinator brought organisations in contact with each other outside the monthly meetings. Fourth, the care coordinator acted as a central contact person for all organisations.
(III) Increase leadership
  In addition to the programme theory, the coordinated partnership strategy was noted to facilitate leadership in three ways (DOC16,27,22; INT3). First, the district managers were the leaders of the monthly meetings. Second, the care coordinators monitored the progress of the approach. They ensured that organisations fulfilled their duties and that they adhered to the plan of action. Third, the care coordinators had a final say in which actions to take and which organisations to involve.

##### Outcomes

In line with the programme theory, the abovementioned mechanisms were described to increase the speed and efficiency with which organisations were able to approach and help the nuisance households (DOC6–8,10–13,16–18,20,23,24; INT1,3,6). Nuisance households were identified more quickly, their background was clear much faster, the speed with which they were helped increased, and duplication of work was prevented.

##### Conditions

(I) Settle privacy issues
  For the coordinated partnership strategy to produce the abovementioned changes it was said to be important to resolve any privacy issues. (DOC15,16; INT1,3,6). Initially, organisations were reluctant to exchange information about households because of privacy issues. A care professional mentioned: “when the care coordinator tried to connect organisations or asked organisations for information, issues of privacy violation and privacy agreements arose (DOC16)”. In response, protocols were established, but these measures were not able to entirely resolve the issue. However, experiences from pilot district 1 suggested that privacy issues may resolve further over time, as organisations got more familiar with the approach and with each other, and as success stories started to emerge.
(II) Include small group of nuisance-oriented organisations as key partners
  For the coordinated partnership strategy to produce abovementioned changes it was mentioned to be important to include a small group of nuisance-oriented organisations as key partners (DOC11,16; INT2-6). Meetings were restricted to the key partners to keep them manageable. Key partners were the organisations with most information on neighbour nuisance and who took most interest in the reduction in neighbour nuisance, i.e. the police and local housing associations. In practice, this meant that care professionals were generally not present during meetings. They were only involved in the execution of the approach.

#### Nuisance households

##### Mechanisms

(I) Stimulate conscience
  In line with the programme theory, the house visit was mentioned to make nuisance households aware of their behaviour and its impact on others (DOC16; INT3). Many households perceived their behaviour to be normal, and were not aware of the consequences of their behaviour for their neighbours and more distant residents. House visits urged nuisance households to reflect on their behaviour, which raised awareness about its impact on others.
(II) Facilitate compliance and control disinhibitors
  In line with the programme theory, assistance was found to help nuisance households solve some of the underlying problems that kept them from changing their behaviour (DOC16,17,23,24; INT1-4,6). This mechanism was mentioned to be essential to achieve long-term changes in nuisance behaviour. Households that had difficulties finding the right assistance for their problems were guided to the right professionals. If households refused to accept the assistance offered to them, they were tempted to use assistance by offering them something in return. For example, when a household was dealing with rent arrears, the housing corporation would be willing to postpone eviction, on the condition that the household would accept assistance.
(III) Set rules
  In line with the programme theory, the guidance agreement was noted to provide nuisance households with a set of rules for the desired behaviour, which urged households to take responsibility for their behaviour (DOC16,17,21; INT3,4,6). In the past, assistance was often offered to nuisance households without setting any rules. For these households, the rules often acted as conditions for assistance. A care manager described:*“more than ever, we call on the own responsibility of the client. Often, these people already have a long history of assistance. Then, it can be necessary to set conditions for the offered assistance. For example, we offer clients a final debt assistance trajectory, under the condition that he cleans his yard* (DOC16)”.
(IV) Increase surveillance
  In addition to the programme theory, the house visit and guidance agreement was mentioned to increase feelings of surveillance among nuisance households (DOC16; INT4,6). Households realised that organisations would keep an eye on them and would approach them when breaking the rules set out in the agreement.
(V) Put pressure
  In addition to the programme theory, threatening with consequences was found to increase the amount of pressure among nuisance households to change their behaviour (DOC16,17,23; INT1,3). This mechanism was only set in motion among a handful of nuisance households for whom the first four mechanisms did not achieve the desired change in nuisance. A programme manager described: “*with some of the five remaining households, we got in touch and told them that it has to change. Otherwise we would, if possible, gnaw at their benefits or evict them. (These are) all measures that you don’t directly have to put into action, but that do apply some pressure* (DOC23)*”*

##### Outcomes

In line with the programme theory, the abovementioned mechanisms were noted to reduce the amount of neighbour nuisance (DOC6-10,13–19,21–23; INT2,3). In 2011, MCN encountered a total of 308 nuisance households in the four target districts (DOC14) (numbers for others years are unknown). Over the years, nearly all nuisance households that were approached by MCN were enrolled in the programme. As a result, the amount of nuisance signals drastically dropped. These outcomes were mainly the result of increased conscience, facilitated compliance, rule setting, and surveillance. A handful of households remained for whom these mechanisms were not sufficient to reduce nuisance. A few of them were successfully targeted with pressure.

##### Conditions

No conditions were identified.

#### Districts

##### Mechanisms

For the four target districts and the city as a whole, Table 3 displays residents’ perceptions of formal and informal social control before and after implementation of MCN (Survey (SURV) 1 and 3, see Table 2 for more details).

**Table 3:** Perceptions of formal and informal social control across districts over time

	Formal social control (%yes)^a^				Informal social control (0 = low;10 = high)^b^			
Districts^c^	2005	2007	2009	2011	2005	2007	2009	2011
Target district 1	22	36	57	52	5.4	4.5	5.0	5.0
Target district 2			63	65			5.6	5.8
Target district 3a			61	49			5.3	5.3
Target district 3b			59	58			5.2	5.3
Target district 3c			60	53			5.2	5.2
Target district 4			52	48			5.1	4.8
City average	21	25	50	50	5.9	5.7	5.8	5.8

^a^Percentage agreeing with the item ‘municipality pays (a lot of) attention to liveability- and safety problems in my neighbourhood’. The question was rephrased from ‘a lot of attention’ in 2005/2007 to ‘attention’ in 2009/2011

^b^Mean score on the items ‘the people in this neighbourhood interact well’, ‘I feel at home with the people living in my neighbourhood’, ‘I live in a nice neighbourhood where there is a lot of solidarity’ and ‘the people in this neighbourhood barely know each other’

^c^MCN was introduced in 2006 in target district 1, and in 2010 in target districts 2 to 4

(I) Perceived formal social control
  In line with the programme theory, perceptions of formal social control improved in districts 1 and 2. In district 1, where MCN was implemented in 2006, the amount of people perceiving much formal social control increased from 22 % in 2005 to 36 % in 2007. In district 2, where MCN was implemented in 2010, numbers increased from 63 % in 2009 to 65 % in 2011. For both districts, changes were more positive than the city average. As opposed to the programme theory, in districts 3 and 4 (where MCN was implemented in 2010) perceptions of formal social control deteriorated between 2009 and 2011. The amount of deterioration ranged from 1 % in target district 3b to 12 % in target district 3a. These changes were more negative than the city average.
(II) Perceived informal social control
  In line with the programme theory, perceptions of informal social control improved in district 2. Mean scores for informal social control increased from 5.6 in 2009 to 5.8 in 2011 (on a scale from 0(low) to 10(high)). This change was more positive than the city average, which did not change over time. As opposed to the programme theory, perceptions of informal social control did not change in district 3 and even deteriorated in districts 1 and 4.

##### Outcomes

For the four target districts and the city as a whole, Table 4 displays residents’ perceptions of neighbour nuisance and general safety before and after implementation of MCN (SURV1-3). In line with the programme theory, perceptions of area safety improved in district 2. The amount of people agreeing that neighbour nuisance occurred often in their neighbourhood decreased from 17 % in 2009 to 12 % in 2011. In the same district, the amount of people agreeing that they sometimes felt unsafe in their neighbourhood decreased from 32 % in 2009 to 30 % in 2011. Both changes were more positive than the city average. As opposed to the programme theory, perceptions of area safety deteriorated in the other districts. These changes were more negative than the city average.

**Table 4:** Perceptions of neighbour nuisance and general safety across districts over time

	Neighbour nuisance (% yes)^a^				General safety (% unsafe)^b^			
Districts^c^	2005	2007	2009	2011	2005	2007	2009	2011
Target district 1	16	17	17	18	35	47	34	37
Target district 2			17	12			32	30
Target district 3a			12	16			31	35
Target district 3b			12	13			28	36
Target district 3c			10	12			30	34
Target district 4			15	17			35	45
City average	12	10	9	9	30	29	25	26

^a^Percentage agreeing with the item ‘neighbour nuisance occurs often in my neighbourhood’

^b^Percentage agreeing with the item ‘I sometimes feel unsafe in my neighbourhood’

^c^MCN was introduced in 2006 in target district 1, and in 2010 in target districts 2 to 4

##### Conditions

(I) Part of wider safety approach
  Interviewees generally agreed that a wider safety approach was a key condition for change (INT1-4,6). District-wide safety perceptions were mentioned to be affected by neighbour nuisance, as well as other problems. District managers talked about how improved safety perceptions were due to an integral approach that consisted of MCN, maintenance of public space, coordinated policing, housing restructuring, and stimulation of local economy. These last two interventions did not only make people feel safer via improved perceptions of public space, but also via gentrification. Housing restructuring and stimulation of local economy were mentioned to attract new residents from higher socio-economic classes. This gentrification process should make people feel safer. In district 1, housing restructuring was found to attract many of its original residents and families from closed communities, which restricted gentrification. In district 2, stimulation of local economy was found to attract new, higher-income residents, which created gentrification. Combined with extra maintenance of public space and coordinated policing, this may explain why positive outcomes were restricted to district 2.
(II) Small and centrally located district
  District 2 is much smaller and much more densely populated than the other districts (Table 1). Some interviewees mentioned a central location within the city of Arnhem and small district size as key conditions for change (INT5,6). District managers suggested that the central location of district 2 within the city of Arnhem facilitated the success of the wider safety approach, particularly by stimulating the local economy.

## Discussion

This realist evaluation study aimed to explore the inner workings of MCN, an area-based intervention to reduce neighbour nuisance in the four most deprived districts of Arnhem, the Netherlands. Results indicate that interventions like MCN are able to reduce neighbour nuisance in deprived areas. By exploring conditions for change, we uncovered why the reduction in neighbour nuisance led to improved perceptions of area safety in some districts but not in others.

### Limitations

This study had some potential limitations that should be taken into account when interpreting the results. According to the realist evaluation method, the impact of the mechanisms that are activated by a programme largely depends on conditions in which they are activated [17]. One of the central aims of a realist evaluation is to identify such conditions. At the district level, we were able to identify several conditions that enabled or constrained the success of MCN, such as population density and the extent to which MCN is integrated into a wider safety approach. However, we had no detailed information on the implementation of MCN in the different districts. As a result, we were limited in our ability to explain between-district differences in effects of MCN on safety perceptions.

We had only limited information available about the intensity of the intervention, including budget and number of organisations involved. Some information about budget was available from a social cost-benefit analysis, which showed that 10 % of the costs for MCN were spent on staff costs for the care coordinators and 90 % was spent on new care trajectories for the nuisance households [26]. However, more detailed information on budget and staffing is needed.

Information on mechanisms and outcomes at the level of nuisance households originated mostly from interviews with district managers and documents from the municipality and other participating organisations. Neighbourhood residents’ views on mechanisms were unknown and their views on the outcome measure were only indirectly included in the form of the number of nuisance signals reported to the authorities. Information about nuisance households’ views on mechanisms and outcomes were absent. We acknowledge that neighbourhood residents and nuisance households might have disclosed alternative views on the mechanisms and outcomes of MCN. More specifically, the participating organisations may have been overly positive about the success of MCN and a more sober picture may have arisen from interviews with household members or their neighbours.

Quantitative data about district level outcomes were obtained from four repeated cross-sectional surveys. Over the years, response rates in the four target districts varied between 24 and 35 %. This is lower than response rates in the city of Arnhem as a whole, and the average response rate for web-based surveys [27]. The low response rate in our districts may have biased our results if non-response was selective in ways related to our study outcome, and if the selectivity of the non-response changed over the years. Unfortunately, we were unable to perform non-response analyses. To reduce some of this bias, survey data was weighted for age and gender and for ethnicity in 2009 and 2011.

### Refining the programme theory

Among participating organisations, the coordinated partnership strategy applied by MCN led to an increase in the efficiency with which households were approached and helped. As suggested by Winer and Karen [23], this outcome may be the result of improved coordination among organisations. Coordination was improved by aligning the roles of the organisations, and by shortening the communication lines. MCN was able to increase efficiency not only because of improved coordination, but also because of improved leadership. This mechanism may explain why power issues have not been a problem with MCN, while Winer and Karen anticipated power issues to be a problem with this strategy [23]. Our results further suggest that two conditions should be met in order for this type of partnership to engender the abovementioned mechanisms. First, privacy issues need to be settled, for example by means of protocols, in order to ensure free exchange of confidential information. Second, it is important to involve only a small group of organisations that are all oriented at the same goal, in our case nuisance reduction.

Among the majority of the nuisance households, the joint assistance and enforcement strategy applied by MCN led to a reduction in nuisance. As suggested by Clarke’s situational crime prevention theory [24], this outcome may be the result of a reduction in the underlying reasons for nuisance, which limited the opportunities for nuisance. Reasons for nuisance were successfully tackled by stimulating conscience, setting rules, facilitating compliance, and controlling disinhibitors. While Clarke suggests using physical strategies (e.g. signs, facilities) to activate these mechanisms, our results show that more socially oriented strategies may be effective as well. Conscience was stimulated by means of the house visit, which made households aware of their behaviour and its impact on others. Rules were set by means of the guidance agreement, which urged households to take responsibility for their behaviour. Compliance was facilitated and disinhibitors were controlled by means of assistance, which helped households to solve problems like debt and addiction. MCN was able to reduce nuisance among most households not only because it reduced the underlying reasons for nuisance but also because it increased feelings of formal surveillance. This mechanism relates to one of Clarke’s other opportunity-reducing measures: increasing risk. In a few households, MCN was unable to reduce nuisance by tackling reasons or increasing perceived surveillance. For some of these families, pressure proved to be a successful mechanism to reduce nuisance. We should note that this mechanism operated in only a small minority of households.

Among residents of one of the four districts, the reduction in neighbor nuisance that was accomplished by MCN led to improved district-wide perceptions of area safety. As suggested by Taylor’s incivilities thesis [25], this outcome may have been the result of improved district-wide perceptions of informal and formal social control. The fact that results were only visible in one of the four districts, suggests that the incivilities thesis only holds under certain conditions. A first condition appears to be that the intervention needs to be part of a wider safety approach. Perceptions of area safety are not only affected by neighbour nuisance, but also by problems like litter, crime, or decay, and by processes like gentrification. A second condition appears to be that the target area needs to be relatively small and centrally located within the city. This seemed to facilitate the success of the wider safety approach. Moreover, we suggest that this type of area may also be more densely populated, as was the case with district 2 (see Table 1), which increases exposure to the nuisance and subsequent actions taken by organisations.

### Methodological considerations

This study illustrates how a realist evaluation can help strengthen the evidence base for complex area-based interventions that aim to improve social determinants of health like area safety. Despite the limitations of our study, we were able to show how and under what conditions a multi-component area-based intervention was able to reduce neighbour nuisance and improve perceptions of area safety. Conventional quantitative evaluation studies would have concluded that MCN has failed, since it was not followed by improved safety perceptions in most target districts. This study shows that these interventions are too complex to judge based on a simple pass or fail verdict, and that more complex evaluation methods like the realist evaluation are needed to understand its complexity. Moreover, this study illustrates how information on mechanisms of change and conditions for success may help refine both the intervention’s programme theory and the theoretical frameworks on which the programme theory is built. However, more realist evaluations are needed to establish a solid evidence base for the health impact of complex area-based interventions, as only a limited amount of information can be retrieved in a single study like ours [28]. Moreover, due to the inherent subjectivity of the realist approach, this type of study needs replication as well as clear standards for analyses and reporting [29].

## Conclusions

This study assessed how area-based interventions like MCN may contribute to population health of deprived areas by improving an environmental determinant of health: area safety. By means of a realist evaluation, we were able to capture the complexity of processes set in motion by interventions like MCN. We gained more insight in the mechanisms by which MCN was able to efficiently and effectively reduce neighbour nuisance. By exploring conditions for change, we understood why the reduction in neighbour nuisance led to improved perceptions of area safety in some districts but not in others. This information may help improve future initiatives elsewhere.

## Availability of data and materials

Most of the data sources described in Table 2 are freely available on the internet, except for the interviews and a few documents. All data sources are available in Dutch only. Questions and requests about the data sources can be directed at the corresponding author.
